# Effects of estimated glomerular filtration rate on clinical outcomes in patients with intracerebral hemorrhage

**DOI:** 10.1186/s12883-022-02551-2

**Published:** 2022-01-10

**Authors:** Zhaoxia Li, Zixiao Li, Qi Zhou, Hongqiu Gu, Yongjun Wang, Xingquan Zhao, on behalf of Chinese Stroke Center Alliance investigators, Kai Wang, Xunming Ji, Xinyue Qin, Ning Wang, Zhaoming Ge, Jinsheng Zeng, Lvli Li, Lan Chu, Zhibin Chen, Li Guo, Guozhong Li, Yuming Xu, Bo Hu, Beisha Tang, Guorong Liu, Xiaoshan Wang, Xiaomu Wu, Yi Yang, Zhiyi He, Zhenhai Wang, Shizheng Wu, Gang Zhao, Meijia Zhu, Qiang Dong, Xiaoyuan Niu, Dong Zhou, Zhongping An, Yuhua Zhao, Xiaoning Zhang, Li Ding, Min Lou

**Affiliations:** 1 Department of Neurology, Beijing Tiantan Hospital, Capital Medical University, Beijing, China; 2 China National Clinical Research Center for Neurological Diseases, Beijing, China; 3 Center of Stroke, Beijing Institute for Brain Disorders, Beijing, China; 4 Beijing Key Laboratory of Translational Medicine for Cerebrovascular Disease, Beijing, China; 5 Research Unit of Artificial Intelligence in Cerebrovascular Disease, Chinese Academy of Medical Sciences, Beijing, China

**Keywords:** Glomerular filtration rate, Chronic kidney disease, Intracerebral hemorrhage, Prognosis, Mortality

## Abstract

**Background:**

The influence of chronic kidney disease (CKD) on the severity and prognosis of spontaneous intracerebral hemorrhage (ICH) has been scarcely investigated. We aimed to explore the association of admission estimated glomerular filtration rate (eGFR) levels with hemorrhagic stroke severity and outcomes in ICH patients.

**Materials and methods:**

The patients enrolled in this study were from the China Stroke Center Alliance study (CSCA). Patients were divided into four groups according to differences in eGFR at admission (≥90; 60–89; 45–59; < 45). Multivariable logistic regression analysis was used to determine the association of the eGFR at admission with hemorrhagic stroke severity, in-hospital complications, discharge disposition, and in-hospital mortality after ICH.

**Results:**

A total of 85,167 patients with acute ICH were included in the analysis. Among them, 9493 (11.1%) had a baseline eGFR<60 ml/min/1.73 m^2^. A low eGFR was associated with an increased risk of in-hospital mortality [eGFR 60–89 ml/min/1.73 m^2^, odds ratio (OR) 1.36 (95% confidence interval (CI) 1.21–1.53); eGFR 45–59, 2.35 (1.97–2.82); eGFR<45, 4.18 (3.7–4.72); *P for trend* < 0.0001], non-routine discharge [eGFR 60–89, 1.11 (1.03–1.2); eGFR 45–59, 1.16 (1–1.35); eGFR<45, 1.37 (1.23–1.53); *P for trend* < 0.0001], hemorrhagic stroke severity [eGFR 60–89, 1 (0.95–1.05); eGFR 45–59, 1.39 (1.26–1.53); eGFR<45, 1.81 (1.67–1.96); *P for trend* < 0.0001], in-hospital complications of pneumonia [eGFR 60–89, 1.1 (1.05–1.14); eGFR 45–59, 1.3 (1.2–1.4); eGFR<45, 1.66 (1.57–1.76); *P for trend* < 0.0001] and hydrocephalus [eGFR 60–89, 0.99 (0.87–1.12); eGFR 45–59, 1.37 (1.1–1.7); eGFR<45, 1.54 (1.32–1.8); *P for trend* = 0.0139] after adjusting for confounding factors. With the decline in eGFR, the risk of hematoma evacuation increased in patients with an eGFR 45 to 59 ml/min/1.73 m^2^ (OR 1.48; 95% CI 1.37–1.61). No significant association between differences in eGFR at baseline and in-hospital complication of recurrent intracerebral hemorrhage was observed.

**Conclusions:**

Low eGFR at baseline was associated with an increased risk of in-hospital mortality, non-routine discharge, hemorrhagic stroke severity and in-hospital complications such as pneumonia, hydrocephalus and hematoma evacuation in acute ICH patients.

## Background

Chronic kidney disease (CKD), defined as a reduced estimated glomerular filtration rate (eGFR) and/or the presence of proteinuria, affects almost 119.5 million Chinese adults aged 18 years or older [1]. Thus, it is becoming a public health problem. Our previous study showed that CKD increased the risk of stroke, including ischemic stroke and hemorrhagic stroke, and all-cause mortality among the Chinese general population [2, 3]. Mechanisms underlying the influence of CKD on the brain are unclear. There are several hypotheses that CKD increases the risk of ischemic stroke by enhancing the process of atherosclerosis, exacerbating platelet dysfunction and aggregation, and activating oxidative stress [4–7]. CKD can also induce volume overload and hypertension through the renin angiotensin aldosterone system, which in turn causes ischemic and hemorrhagic stroke [8, 9].

The prevalence of spontaneous intracerebral hemorrhage (ICH) is high in China and carries substantial risk for disability and mortality [10, 11]. Most of ICH cases are due to hypertension [11]. Renal and brain perforating arteries are short, small arteries, so the mechanisms through which perfusion pressure and blood flow are maintained are similar [8, 12]. Therefore, both ICH and CKD can be attributed to small vessel disease. A decreasing glomerular filtration rate (GFR) affects not only nephron arteries but also cerebral arteries [12]. CKD increases the risk of worse outcomes, stroke severity, and hemorrhagic transformation among ischemic stroke patients, which has been elucidated in some studies [13–15]. However, there are few studies exploring the relationship between CKD and outcomes of ICH, especially with large sample sizes from Asian populations.

The aim of this study was therefore to assess the relationship between different levels of eGFR and in-hospital mortality, hemorrhagic stroke severity, discharge disposition, and in-hospital complications among ICH patients from the China Stroke Center Alliance.

## Materials and methods

### Study design and participants

The data for this study were obtained from the China Stroke Center Alliance (CSCA). Details of the study design have been described previously [16]. Briefly, the study was a national, hospital-based, multicenter, voluntary, multifaceted intervention and continuous quality improvement initiative performed in China. The study was approved by the Chinese Stroke Center Alliance, the Beijing Tiantan Hospital Ethics Committee (the ethical reference number is KY 2018–061-02), in accordance with the Declaration of Helsinki. All methods were carried out in accordance with relevant guidelines and regulations. Participating hospitals received a healthcare quality assessment and research approval to collect data in the CSCA without requiring individual patient informed consent under the common rule or a waiver of authorization and exemption from their Institutional Review Board. Patient informed consent was waived by the Beijing Tiantan Hospital Ethics Committee. Patient confidentiality was protected in the following ways: (1) data were stripped of all identifiers before their use in research, and (2) the use of data for these purposes is closely overseen by the China National Clinical Research Center for Neurological Diseases analytic center [16]. Between August 2015 and July 2019, 1,006,798 consecutive patients aged 18 years or older with acute stroke or transient ischemic attack (TIA) confirmed by brain computed tomography (CT) magnetic resonance imaging (MRI) within 7 days of symptom onset across 1312 designed hospitals in China were included. Among the trials, 85,705 patients were diagnosed with spontaneous ICH. A total of 538 patients were excluded due to missing data on serum creatinine levels. Therefore, 85,167 patients, including 53,208 men and 31,959 women, were ultimately included in this analysis.

### Demographic and clinical information

Demographic characteristics, medical history and laboratory data were collected at admission. Body mass index (BMI) was calculated as kg/m^2^. Hypertension was classified as blood pressure ≥ 140/90 mmHg, self-reported history of hypertension, or antihypertensive medication use. Diabetes mellitus was defined by self-reported history, use of hypoglycemic medications, or fasting glucose level ≥ 7.0 mmol/l. Hypercholesterolemia was defined as a self-reported history or use of lipid-lowering medication. Current smoking was defined as smoking more than one cigarette a day. Alcohol use was defined as drinking more than three glasses of wine (or equivalent alcohol) per day. The severity of stroke was assessed using the Glasgow Coma Scale (GCS) and National Institutes of Health Stroke Scale (NIHSS). In-hospital mortality and complications (including pneumonia, pulmonary embolism, urinary tract infection, seizure, hydrocephalus, recurrent intracerebral hemorrhage, gastrointestinal bleeding, and deep vein thrombosis (DVT)), hematoma evacuation, length of hospital stay, hospital expenditure, and discharge disposition were recorded.

### Estimation of glomerular filtration rate and measurement of kidney function

Baseline serum creatinine (SCr) was measured by an automated hematology analyzer at each research center using the enzymatic method. GFR was estimated by using a modified 4-variable Chronic Kidney Disease Epidemiology Collaboration (CKD-EPI) formula with an adjusted coefficient of 1.1 for the Chinese population [17]: eGFR_CKD-EPI_ = 141 × min (SCr/κ,1)^α^ × max (SCr/κ,1)^− 1.209^ × 0.993^Age^ × 1.018 (if female) × 1.1, where SCr was serum creatinine, κ was 0.7 for females and 0.9 for males, α was − 0.329 for females and − 0.411 for males, min was the minimum of SCr/κ or 1, and max indicated the maximum of SCr/κ or 1. The eGFR values were divided into four categories, < 45, 45 to 59, 60 to 89, and ≥ 90 ml/min/1.73 m^2^, which were based on the National Kidney Foundation’s Kidney Disease Outcomes Quality Initiative (NKFK/DOQI )[18].

### Outcomes

The primary outcome was in-hospital mortality. The secondary outcomes included hemorrhagic stroke severity, discharge deposition, and in-hospital complications. Severe hemorrhagic stroke was defined as NIHSS≥11. Patients who discharge home represents that the patient’s condition is stable, we consider it as a routine discharge. While patients who discharge to a grade II or III hospital, community hospital, or rehabilitation facility means that they need more care, and we consider it as a non-routine discharge. The in-hospital complications included pneumonia, pulmonary embolism, urinary tract infection, seizure, hydrocephalus, hematoma evacuation, recurrent intracerebral hemorrhage, gastrointestinal bleeding, and DVT [16].

### Statistical analysis

Categorical variables are presented as frequencies with percentages and were compared using the chi-square test. Continuous variables were assessed for a normal distribution using the Kolmogorov–Smirnov test. Normally distributed data are described as the mean ± standard deviation and were compared using one-way ANOVA. Skew distributed data are described by medians with interquartile ranges and were compared using the Mann–Whitney U test. Logistic regression models were performed to calculate the odds ratios and 95% confidence intervals for the association between eGFR and in-hospital mortality, hemorrhagic stroke severity, in-hospital complications, and discharge disposition. Model 1 was adjusted for age and sex. Model 2 was adjusted for age, sex, BMI, current smoking, prior stroke or TIA, prior chronic heart disease (CHD) or myocardial infarction, hypertension, dyslipidemia, atrial fibrillation, diabetes mellitus, peripheral vascular disorder (PVD), alcohol consumption. A two-sided *p* value < 0.05 was considered to be statistically significant. Statistical analyses were performed using SAS software version 9.4 (SAS Institute Inc., Cary, NC, USA).

## Results

A total of 85,705 patients diagnosed with spontaneous ICH from 1312 designed hospitals in China were enrolled in CSCA. We excluded 538 patients whose records lacked serum creatinine data. Finally, 85,167 patients with acute ICH were included in the analysis.

Table 1 and Table 2 show the demographic and clinical characteristics of ICH patients according to differences in eGFR. The mean age was 62.9 years, and 62.5% (*n* = 53,208) were men. The most prevalent ICH risk factor was hypertension (72.2%, *n* = 61,488). At hospital admission, the median serum creatinine was 67.7 μmol/L, the median eGFR was 101.4 ml/min/1.73 m^2^, and 9493 (11.1%) patients had an eGFR less than 60 ml/min/1.73 m^2^. A total of 58,418 patients (68.6%) had an eGFR≥90, 17,256 (20.3%) had an eGFR 60 to 89, 3507 (4.1%) had an eGFR45 to 59, and 5986 (7.0%) had an eGFR< 45 mL/min/1.73 m^2^. The median NIHSS score at admission was 6 (interquartile range 2 to 12). A total of 1975 (2.3%) patients died in hospital.

**Table 1:** Clinical characteristics among intracerebral hemorrhage patients grouped by baseline estimated glomerular filtration rate

Variables	eGFR at baseline (ml/min/1.73 m^**2**^)					
	Total (***N*** = 85,167)	≥90 (***N*** = 58,418)	60–89 (***N*** = 17,256)	45–59 (***N*** = 3507)	< 45 (***N*** = 5986)	***P*** Value
**Demographic**						
Age, y, mean (SD)	62.9 ± 12.9	60.8 ± 12.1	68.3 ± 12.9	69.5 ± 13.4	63.8 ± 13.9	<.0001
Male, n (%)	53,208 (62.5)	36,846 (63.1)	10,680 (61.9)	2060 (58.7)	3622 (60.5)	<.0001
**Physical examination, mean (SD)**						
BMI, kg/m^2^	23.9 ± 4.5	23.9 ± 4.1	23.6 ± 4.3	23.6 ± 3.9	24.3 ± 8.2	<.0001
SBP, mmHg	164.6 ± 28.2	163.3 ± 27.34	166.3 ± 28.9	169.0 ± 30.1	168.7 ± 32.	<.0001
DBP, mmHg	95.3 ± 16.9	95.2 ± 16.4	94.8 ± 17.4	95.7 ± 18.2	97.1 ± 19.3	<.0001
**Medical history, n (%)**						
Prior stroke or TIA	24,472 (28.7)	16,177 (27.7)	5096 (29.5)	1128 (32.2)	2071 (34.6)	<.0001
Prior CHD or myocardial infarction	4779 (5.6)	2945 (5.0)	1136 (6.6)	293 (8.4)	405 (6.8)	<.0001
Hypertension	61,488 (72.2)	41,188 (70.5)	12,892 (74.7)	2706 (77.2)	4702 (78.5)	<.0001
Dyslipidemia	7246 (8.5)	4636 (7.9)	1613 (9.3)	370 (10.6)	627 (10.5)	<.0001
Atrial fibrillation	1304 (1.5)	644 (1.1)	425 (2.5)	111 (3.2)	124 (2.1)	<.0001
Diabetes mellitus	8335 (9.8)	5182 (8.9)	1731 (10.0)	446 (12.7)	976 (16.3)	<.0001
PVD	822 (1.0)	465 (0.8)	205 (1.2)	46 (1.3)	106 (1.8)	<.0001
**Behavioral history, n (%)**						
Current smoking	16,767 (19.7)	12,179 (20.8)	3080 (17.8)	556 (15.9)	952 (15.9)	<.0001
Drinking	20,790 (24.4)	14,795 (25.3)	3812 (22.1)	705 (20.1)	1478 (24.7)	<.0001
**Laboratory test, median (IQR)**						
LDL cholesterol, mmol/L	2.6 (2.0–3.2)	2.6 (2.0–3.2)	2.6 (2.1–3.2)	2.7 (2.1–3.4)	2.8 (1.9–4.3)	<.0001
GHb, mmol/L	139.0 (125.0–153.0)	141.0 (128.0–154.0)	134.0 (121.0–149.0)	130.0 (120.0–143.0)	120.0 (100.0–143.0)	<.0001
Hcy, mmol/L	13.7 (10.0–20.0)	13.0 (9.5–19.0)	14.6 (10.5–20.6)	16.0 (11.3–23.1)	18.9 (10.5–31.5)	<.0001
Creatinine, μmmol/L	67.7 (55.0–85.0)	60.0 (50.1–70.0)	88.0 (77.0–98.8)	118.3 (101.6–131.0)	231.0 (159.0–440.0)	<.0001
BUN, mmol/L	5.1 (4.0–6.6)	4.8 (3.8–6.0)	5.8 (4.7–7.2)	7.1 (5.7–9.0)	9.1 (5.3–13.1)	<.0001
eGFR, ml/min/1.73 m^2^	101.4 (84.0–113.6)	108.8 (100.5–118.3)	78.6 (70.9–85.0)	53.9 (49.9–57.2)	23.4 (10.4–35.0)	<.0001
Uric acid, μmmol/L	277.0 (210.0–354.0)	260.0 (200.4–329.0)	311.0 (240.7–382.5)	347.0 (263.0–432.0)	352.0 (216.0–483.0)	<.0001

*SD* standard deviation; *BMI* body mass index; *SBP* systolic blood pressure; *DBP* diastolic blood pressure; *TIA* transient ischemic attack; *CHD* chronic heart disesea; *PVD* peripheral vascular disorder; *LDL* low-density lipoprotein; *GHb* glycated hemoglobin; *Hcy* homocysteine; *BUN* blood urea nitrogen; *eGFR* estimated glomerular filtration rate

**Table 2:** Clinical characteristics and severity and in-hospital outcome in ICH patients

Variables	eGFR at baseline (ml/min/1.73 m^**2**^)					
	Total (***N*** = 85,167)	≥90 (***N*** = 58,418)	60–89 (***N*** = 17,256)	45–59 (***N*** = 3507)	< 45 (***N*** = 5986)	***P*** Value
**In hospital NIHSS, median (IQR)**	6.0 (2.0–12.0)	5.0 (2.0–12.0)	6.0 (2.0–12.0)	7.0 (3.0–14.0)	8.0 (3.0–18.0)	<.0001
**Hemorrhagic stroke severity by NIHSS**						<.0001
Missing	36,155	24,376	7144	1545	3090	
Score 0–5, n (%)	21,264 (43.4)	15,070 (44.3)	4463 (44.1)	757 (38.6)	974 (33.6)	
Score 6–10, n (%)	13,537 (27.6)	9599 (28.2)	2740 (27.1)	495 (25.2)	703 (24.3)	
Score ≥ 11, n (%)	14,211 (29.0)	9373 (27.5)	2909 (28.8)	710 (36.2)	1219 (42.1)	
**GCS, median (IQR)**	13.0 (8.0–15.0)	14.0 (8.0–15.0)	13.0 (8.0–15.0)	12.0 (7.0–15.0)	11.0 (6.0–15.0)	<.0001
**In-hospital mortality, n(%)**	1975 (2.3)	955 (1.6)	437 (2.5)	157 (4.5)	426 (7.1)	<.0001
**In-hospital complication, n(%)**						
Pneumonia	21,795 (25.6)	13,673 (23.4)	4883 (28.3)	1132 (32.3)	2107 (35.2)	<.0001
Pulmonary embolism	229 (0.3)	149 (0.3)	52 (0.3)	9 (0.3)	19 (0.3)	0.6504
Urinary tract infection	2106 (2.5)	1386 (2.4)	457 (2.6)	85 (2.4)	178 (3.0)	0.0123
Seizure	1191 (1.4)	808 (1.4)	234 (1.4)	50 (1.4)	99 (1.7)	0.3654
DVT	1125 (1.3)	761 (1.3)	242 (1.4)	45 (1.3)	77 (1.3)	0.7719
Gastrointestinal bleeding	1014 (1.2)	728 (1.2)	195 (1.1)	37 (1.1)	54 (0.9)	0.0767
Hydrocephalus	1862 (2.2)	1216 (2.1)	352 (2.0)	98 (2.8)	196 (3.3)	<.0001
Hematoma evacuation Recurrent intracerebral hemorrhage	8901 (10.5) 7026 (8.2)	6258 (10.7) 4762 (8.2)	1473 (8.5) 1381 (8.0)	326 (9.3) 316 (9.0)	844 (14.1) 567 (9.5)	<.0001 0.008
**Length of hospital stay, mean (SD)**	16.6 ± 11.7	17.0 ± 11.6	16.0 ± 11.3	15.5 ± 12.0	15.1 ± 12.6	<.0001
**Hospital expenditure RMB, mean (SD)**	18,399.1 ± 17,035.9	18,425.3 ± 16,947.2	17,540.2 ± 16,139.2	18,435.6 ± 17,117.9	20,627.6 ± 19,959.9	<.0001
**Non-routine discharge**, n (%)	4952 (5.8)	3318 (5.7)	979 (5.7)	208 (5.9)	447 (7.5)	<.0001

*NIHSS* National Institutes of Health Stroke Scale; *GCS* Glasgow coma scale; *DVT* deep vein thrombosis

Table 1 demonstrates that compared with those with a normal eGFR, patients with a decreased eGFR were older, more likely to be female, had a higher prevalence of BMI, SBP and DBP, and had a higher burden of vascular risk factors and comorbidities, including a history of stroke or TIA, coronary artery disease or myocardial infarction, hypertension, dyslipidemia, atrial fibrillation, diabetes mellitus and PVD, but they were less likely to be current smokers or drinkers (*P*<0.001).

Table 2 shows that patients in the lowest eGFR category had higher NIHSS scores and lower Glasgow Coma Scale (GCS) scores; were more likely to have severe hemorrhagic stroke; were more prone to die in the hospital; were more likely to have in-hospital complications, including pneumonia, hydrocephalus, hematoma evacuation and recurrent intracerebral hemorrhage; had higher hospital expenditure; and were more likely to be discharged to a grade II/III hospital, community hospital or rehabilitation facility instead of home but had a shorter length of hospital stay than those in the highest eGFR category (*P*<0.001). There were no significant differences in complications such as pulmonary embolism, urinary tract infection, seizure, DVT, or gastrointestinal bleeding among the groups (all *P* > 0.05).

The associations between eGFR and in-hospital mortality, discharge disposition, hemorrhagic stroke severity and in-hospital complications were further explored using logistic regression analysis (Table 3). In unadjusted logistic regression analysis, the risk of in-hospital mortality, non-routine discharge, hemorrhagic stroke severity and in-hospital complications of pneumonia and hydrocephalus increased sharply as the eGFR declined. After adjusting for all the possible confounders, eGFR less than 60 ml/min/1.73 m^2^ remained to be an independent factor for in-hospital mortality [eGFR 60–89, 1.36 (1.21–1.53); eGFR 45–59, 2.35 (1.97–2.82); eGFR<45, 4.18 (3.7–4.72); *P for trend* < 0.0001], non-routine discharge [eGFR 60–89, 1.11 (1.03–1.2); eGFR 45–59, 1.16 (1–1.35); eGFR<45, 1.37 (1.23–1.53); *P for trend* < 0.0001], hemorrhagic stroke severity [eGFR 60–89, 1 (0.95–1.05); eGFR 45–59, 1.39 (1.26–1.53); eGFR<45, 1.81 (1.67–1.96); *P for trend* < 0.0001] and in-hospital complications of pneumonia[eGFR 60–89, 1.1 (1.05–1.14); eGFR 45–59, 1.3 (1.2–1.4); eGFR<45, 1.66 (1.57–1.76); *P for trend* < 0.0001] and hydrocephalus [eGFR 60–89, 0.99 (0.87–1.12); eGFR 45–59, 1.37 (1.1–1.7); eGFR<45, 1.54 (1.32–1.8); *P for trend* = 0.0139].

**Table 3:** Logistic regression of the eGFR levels on in-hospital mortality and discharge disposition

	Baseline eGFR (mL/min/1.73 m^**2**^)				
	≥90(n = 58,418)	60–89(N = 17,256)	45–59(N = 3507)	< 45(N = 5986)	P for trend
**In-hospital mortality(*N*** **= 1975)**	*N* = 955	*N* = 437	*N* = 157	*N* = 426	
Unadjusted model OR(95% CI)	Ref.	1.56 (1.39–1.75)	2.82 (2.37–3.35)	4.62 (4.1–5.19)	< 0.001
Model 1 OR(95% CI)	Ref.	1.36 (1.21–1.53)	2.41 (2.02–2.88)	4.38 (3.89–4.93)	< 0.001
Model 2 OR(95% CI)	Ref.	1.36 (1.21–1.53)	2.35 (1.97–2.82)	4.18 (3.7–4.72)	< 0.001
**Non-routine discharge (*N*** **= 4952)**	*N* = 3318	*N* = 979	*N* = 208	*N* = 447	
Unadjusted model OR(95% CI)	Ref.	1 (0.93–1.07)	1.11 (1.03–1.2)	2.15 (1.31–3.55)	< 0.001
Model 1 OR(95% CI)	Ref.	1.05 (0.91–1.21)	1.19 (1.03–1.38)	4.17 (1.86–9.37)	< 0.001
Model 2 OR(95% CI)	Ref.	1.11 (1.03–1.2)	1.16 (1–1.35)	1.37 (1.23–1.53)	< 0.001
**Severe hemorrhagic stroke(*N*** **= 14,211)**	*N* = 9373	*N* = 2909	*N* = 710	*N* = 1219	
Unadjusted model OR(95% CI)	Ref.	1.06 (1.01–1.12)	1.49 (1.36–1.64)	1.91 (1.77–2.07)	< 0.001
Model 1 OR(95% CI)	Ref.	0.99 (0.94–1.04)	1.37 (1.24–1.51)	1.85 (1.71–2)	< 0.001
Model 2 OR(95% CI)	Ref.	1 (0.95–1.05)	1.39 (1.26–1.53)	1.81 (1.67–1.96)	< 0.001
**In-hospital complication**					
**Pneumonia(*N*** **= 21,795)**	*N* = 13,673	*N* = 4883	*N* = 1132	*N* = 2107	
Unadjusted model OR(95% CI)	Ref.	1.29 (1.24–1.34)	1.56 (1.45–1.68)	1.78 (1.68–1.88)	< 0.001
Model 1 OR(95% CI)	Ref.	1.1 (1.06–1.15)	1.31 (1.21–1.41)	1.69 (1.59–1.78)	< 0.001
Model 2 OR(95% CI)	Ref.	1.1 (1.05–1.14)	1.3 (1.2–1.4)	1.66 (1.57–1.76)	< 0.001
**Hydrocephalus(*N*** **= 1862)**	*N* = 1216	*N* = 352	*N* = 98	*N* = 196	
Unadjusted model OR(95% CI)	Ref.	0.98 (0.87–1.1)	1.35 (1.1–1.67)	1.59 (1.37–1.86)	< 0.001
Model 1 OR(95% CI)	Ref.	0.99 (0.88–1.12)	1.37 (1.11–1.69)	1.6 (1.37–1.86)	< 0.001
Model 2 OR(95% CI)	Ref.	0.99 (0.87–1.12)	1.37 (1.1–1.7)	1.54 (1.32–1.8)	0.0139
**Recurrent intracerebral hemorrhage(*N*** **= 7026)**	*N* = 4762	*N* = 1381	*N* = 316	*N* = 567	
Unadjusted model OR(95% CI)	Ref.	0.98 (0.92–1.04)	1.12 (0.99–1.26)	1.18 (1.08–1.29)	0.001
Model 1 OR(95% CI)	Ref.	0.97 (0.91–1.03)	1.1 (0.98–1.24)	1.17 (1.07–1.29)	0.001
Model 2 OR(95% CI)	Ref.	0.96 (0.89–1.02)	1.02 (0.9–1.16)	1.07 (0.97–1.18)	0.489
**Hematoma evacuation(*N*** **= 8901)**	*N* = 6258	*N* = 1473	*N* = 326	*N* = 844	
Unadjusted model OR(95% CI)	Ref.	0.78 (0.73–0.83)	0.85 (0.76–0.96)	1.37 (1.27–1.48)	< 0.001
Model 1 OR(95% CI)	Ref.	0.93 (0.88–0.99)	1.05 (0.93–1.18)	1.46 (1.35–1.58)	< 0.001
Model 2 OR(95% CI)	Ref.	0.94 (0.88–1) 1.06 (0.94–1.2)		1.48 (1.37–1.61)	0.0247

Model 1: Adjusted for age, sex

Model 2: Adjusted for age, sex, BMI, current smoking, Prior stroke or TIA, Prior CHD or myocardial infarction, Hypertension, Dyslipidemia, Atrial fibrillation, Diabetes mellitus, PVD, Alcohol consumption

Compared to patients with an eGFR ≥90 ml/min/1.73 m^2^, patients with an eGFR of less than 45 ml/min/1.73 m^2^ had an increased risk of hematoma evacuation (OR1.48; 95% CI 1.37–1.61) after adjusting for confounding factors.

In the crude model and model 1, which was adjusted for age and sex, there was trend showing that with the decline in eGFR, the risk of recurrent intracerebral hemorrhage increased. However, the relationship disappeared after adjusting for other confounders.

## Discussion

In this study, we found that reduced eGFR at baseline was associated with an increased risk of in-hospital mortality, non-routine discharge, hemorrhagic stroke severity and in-hospital complications such as pneumonia, hydrocephalus, and hematoma evacuation in acute ICH patients.

Few studies have explored the association between eGFR and adverse outcomes among ICH patients, and the results have been controversial [19–22]. In a large cohort study of 113,059 patients hospitalized across 1472 United States centers, ICH with renal dysfunction was strongly related to inpatient mortality [19]. In a small sample size prospective study including 365 patients with ICH, after a 3-month follow-up, patients with low eGFR at baseline had an increased risk of all-cause mortality [20]. A study of 1758 acute stroke patients, including 566 hemorrhagic stroke patients admitted to a hospital in China, revealed that decreased eGFR was an independent predictor of death/disability in hemorrhagic stroke patients but not ischemic stroke patients [21]. Our results are in accordance with these studies. Another study from China enrolled 1909 patients with acute stroke, including ICH, and found that a low eGFR had no relationship with an increased risk of death/disability at 3 months [22]. This discrepancy may be attributable to the differences in the study populations and study design.

The mechanisms behind how a low eGFR impacts ICH remain unexplored. However, several explanations can be proposed for the link between CKD and adverse outcomes in patients with stroke. First, a decline in eGFR leads to electrolyte imbalances, causing vasoconstriction and increase blood pressure by the action of aldosterone on sodium-water retention [8, 9]. Second, renal dysfunction increases the bleeding tendency due to platelet dysfunction [23]. Third, CKD has been associated with inflammation and endothelial dysfunction, which may accelerate leukocyte infiltration and further contribute to arteriosclerosis and platelet dysfunction [7]. Together, these factors contribute to hematoma expansion, hemorrhagic transformation, and cerebral microbleeds and lead to adverse outcomes among stroke patients. Several studies have found that patients with moderate/severe kidney impairment had larger hematoma volumes and unfavorable outcomes [24–26]. In an analysis of 770 participants with ischemic stroke, a low eGFR was independently associated with a high risk of hemorrhagic transformation after ischemic stroke [27]. The association of CKD with cerebral microbleeds has been reported in some studies, which reinforces the notion of a link between hypertensive vasculopathy, renal impairment and stroke [28–30]. It is a pity that in our study we did not collect the data of hematoma volume. Yet, our study shows that low eGFR was an independent indicator for severe hemorrhagic stroke and hematoma evacuation. There was trend showing that with a decline in eGFR, the risks of recurrent intracerebral hemorrhage increased; however, those relationships disappeared after adjusting for confounders.

The in-hospital mortality is 2.3% in our study, which is much lower than the other studies in other countries [31]. The low mortality rate may be attributed to the following reasons. Firstly, the CSCA design excluded of early out-of-hospital deaths and emergency department death. Secondly, due to the cultural differences and economic reasons, many patients withdraw from treatment and discharge against medical advice (DAMA) because of their severe condition. This may jointly underestimate the mortality of ICH in our study. In our newly published study, we regard patients who leave the hospital against medical advice or in-hospital death as a major poor outcome, and found that the in-hospital death or DAMA is up to 17.2% in ICH [32].

Our study provides insight on the mortality, discharge dispositions and in-hospital complications of eGFR in ICH based on a large prospective registration with national representation. It supports that low eGFR is an unfavorable outcome predictor in Asian patients with acute ICH. There were several limitations in our study that should be mentioned. First, the measurement of serum creatinine was performed locally rather than at a central laboratory and was not calibrated across laboratory sites, which may have produced substantial variability in the measured values. Second, data were ascertained from patient medical records, and their accuracy depended on the completeness of clinical documentation. Third, we were unable to assess the effect of proteinuria on acute ICH due to a lack of data, even though proteinuria has been shown to be an important independent risk factor for ischemic stroke [33–35]. Fourth, although we adjusted for known confounders, potential sources of confounding factors could have affected our results. Fifth, we did not collect the variables of hematoma volume and locations of hematoma, thus we cannot analyze the effect of CKD on hematoma volume and locations of hematoma, which may have an influence on it. Finally, due to the lack of follow-up data, we were unable to assess the long-term impact of CKD on ICH-related outcomes. Further prospective and multicenter evaluations are necessary to verify the results of this study.

## Conclusions

Reduced eGFR at baseline was associated with an increased risk of in-hospital mortality, non-routine discharge, hemorrhagic stroke severity, and in-hospital complications such as pneumonia, hydrocephalus, hematoma evacuation in acute ICH patients. It was also found to be an independent factor affecting the prognosis of patients with ICH.

## Data Availability

Due to CSCA project regulations, data that support the findings of this study is not publicly available. If someone wants to request the data, please contact the investigators of the Beijing tiantan hospital with reasonable request.

## Abbreviations

CKD: Chronic kidney disease
ICH: Intracerebral hemorrhage
eGFR: Estimated glomerular filtration rate
CSCA: China Stroke Center Alliance study
OR: Odds ratios
CI: Confidence interval
TIA: Transient ischemic attack
CT: Computed tomography
MRI: Magnetic resonance imaging
BMI: Body mass index
GCS: Glasgow Coma Scale
NIHSS: National Institutes of Health Stroke Scale
DVT: Deep vein thrombosis
SCr: Serum creatinine
CKD-EPI: Chronic kidney disease epidemiology collaboration
NKFK/DOQI: National Kidney Foundation’s Kidney Disease Outcomes Quality Initiative
SBP: Systolic blood pressure
DBP: Diastolic blood pressure
CHD: Chronic heart disease
PVD: Peripheral vascular disorder
LDL: Low-density lipoprotein
GHb: Glycated hemoglobin
HCY: Homocysteine
BUN: Blood urea nitrogen
